# A Closed-Form Analytical Evaluation of the Directivity of Planar Array Antennas with Different Types of Radiating Elements

**DOI:** 10.3390/s26175350

**Published:** 2026-08-24

**Authors:** Margaux Pellet, Piero Angeletti, Giovanni Toso

**Affiliations:** European Space Agency, 2200 AZ Noordwijk, The Netherlands; piero.angeletti@esa.int (P.A.); giovanni.toso@esa.int (G.T.)

**Keywords:** active antenna arrays, planar antenna arrays, directivity

## Abstract

This work presents a fast and general formulation for the directivity computation of planar antenna arrays composed of radiating elements modeled as $\cos^{q}\theta$ transmitters. The main novelty in the proposed formulation as compared to the literature is the fact that an arbitrary number of different radiating elements, each one characterized by a different q-factor, can be considered. The proposed framework enables the evaluation and validation of array architectures previously introduced in the literature, including concentric-ring configurations with tapered element sizes. A unified expression is derived in which the directivity depends solely on two inputs: the array factor and the class-dependent radiating element size. This reduced representation allows real-time computation of directivity, even for electrically large planar arrays comprising multiple rings and heterogeneous element classes. The resulting computational complexity is significantly lower than that of classical integral-based approaches traditionally used for directivity evaluation. Numerical results confirm that the proposed formulation yields accurate directivity values that are fully consistent with existing analytical and numerical results reported in the literature.

## 1. Introduction

The directivity of phased arrays is a fundamental performance indicator in modern communication, radar, and sensing systems. It quantifies the ability of an array to radiate energy in a desired direction relative to an isotropic radiating element. Although conceptually straightforward, the computation of directivity for electrically large or irregular arrays remains a significant challenge. Classical formulations require evaluating the total radiated power through a surface integral of the squared magnitude of the radiated field over the entire sphere. For an array of *N* elements, the radiated field is expressed as a coherent summation of all element contributions, leading to a double summation over element indices combined with a double angular integration over (θ,ϕ). As a result, the overall computational complexity scales on the order of $O(N^{2})$ even before performing the angular integration, making real-time or iterative directivity evaluation computationally prohibitive for large arrays.

This difficulty becomes particularly limiting in applications requiring fast pattern evaluation, such as adaptive beamforming, onboard calibration loops, array optimization, or any scenario involving repeated radiation-pattern computations. The computational load is further exacerbated when embedded element patterns are non-isotropic or when arrays exhibit irregular geometries for which analytical simplifications are not applicable.

A wide range of methods have been proposed in the literature to mitigate the computational complexity associated with directivity evaluation. Early approaches relied on far-field approximations or pattern-factorization techniques that exploit aperture separability [1,2,3,4], thus simplifying the radiation integrals but largely restricting their use to highly symmetric or uniformly spaced arrays [5,6,7,8]. In 1970, analytical formulations were proposed based on very general considerations, therefore keeping a significant but limited complexity reduction [1]. Subsequent developments introduced analytical bounds and asymptotic approximations, including stationary-phase evaluations and Fourier-transform-based interpretations of the array factor [9,10,11,12,13]. These methods provide faster estimates of directivity but may lose accuracy when modeling near-in sidelobes or non-uniform excitations [3,14]. More recent research has shifted toward numerical acceleration strategies, such as FFT-based pattern computation, adaptive quadrature schemes, and sparsity-aware compression of the array factor, all aimed at reducing the inherent $O(N^{2})$ scaling of classical formulations [15,16,17,18,19]. Complementing these numerical techniques, several studies have sought closed-form or semi-closed-form directivity expressions for particular classes of element patterns or array geometries. Among these, the matrix-based quadratic-form model proposed in [1] for linear arrays represents an important advance by replacing field integrals with algebraic operations. However, such formulations have so far remained limited to one-dimensional arrays or to configurations using a single class of elements [20], leaving open the need for a general and computationally efficient framework applicable to heterogeneous two-dimensional planar arrays [21].

To reduce the complexity of the electromagnetic field representation [22,23], many large-array models approximate individual element patterns using functions of the form $\cos^{q}\theta$. These patterns provide an analytically tractable means of representing the effects of element size, aperture distribution, and power spreading while avoiding the need for full-wave simulations or complex geometric modeling. Their simplicity enables closed-form expressions for intermediate quantities and makes them attractive for real-time or large-scale array computations.

In ref. [21], the directivity of a linear array composed of a single class of $\cos^{q}\theta$ radiating elements was investigated. The authors introduced an elegant matrix-based quadratic-form representation that replaces the classical field integral with a more compact algebraic expression. This formulation yields significant computational savings and provides direct insight into the dependence of directivity on array geometry and excitation.

Building on the theoretical foundation of [14,21], subsequent work has explored array architectures that exploit element tapering or geometric shaping to achieve desirable radiation characteristics. In particular, planar arrays composed of concentric rings have been proposed, wherein element sizes follow a Taylor-like tapering law: central rings host smaller elements, while outer rings contain progressively larger radiating elements. This configuration results in excellent control of near-in and far-out sidelobes. Variants of this architecture impose constraints on the element sizes in the outer rings to improve manufacturability or satisfy system-level constraints while maintaining favorable sidelobe behavior.

The present work extends these contributions in two important directions. First, a general formulation is derived for the directivity of arrays composed of multiple classes of radiating elements whose embedded element patterns follow $\cos^{q_{n}}\theta$. This extension is critical for accurately modeling non-uniform or radially tapered array architectures. This formulation is validated against already existing formulations and tools. Secondly, the derivation generalizes the one-dimensional quadratic-form framework of [24] to the full two-dimensional case, where elements are arbitrarily placed in the (x,y) plane and the radiation pattern depends on both azimuth ϕ and elevation θ. The proposed formulation eliminates the need for explicit surface integrals of the radiated field, offering a substantial computational advantage over classical methods [25].

Overall, this work provides a unified, and computationally efficient framework for evaluating the directivity of planar arrays composed of multiple element classes. The resulting expressions are well suited for large-scale arrays and real-time computations in next-generation antenna systems, including satellite communications, high-resolution radar, remote sensing, and emerging 6G or non-terrestrial network applications.

## 2. Materials and Methods

### 2.1. System Model: Two Types of cos^q^ Radiating Elements

Consider a planar antenna array composed of *N* radiating elements. Each element belongs to one of two radiating element classes:

- Class 1 elements have the embedded element pattern $E_{1}\left(\theta \right)=\cos^{q_{1}}\theta$;
- Class 2 elements have $E_{2}\left(\theta \right)=\cos^{q_{2}}\theta .$

Let $I_{1},\;I_{2}\subset \left\{1,\ldots ,N\right\}$ be index sets of elements of type 1 and type 2, with $|I_{1}|=N_{1},\;\left|I_{2}\right|=N_{2},\;N_{1}+N_{2}=N$.

Each element *i* is located at position $(x_{i},y_{i}),$ and has complex weight $w_{i}$.

The 2D array manifold factor for element *i* at (θ,ϕ) is given in Equation (1).

(1) $$\nu_{i}\left(\theta ,\phi \right)=e^{jk(x_{i}\sin\theta \cos\phi +y_{i}\sin\theta \sin\phi )}.$$

Therefore the total array factor is defined in (2).

(2) $$\begin{array}{cc} f(\theta ,\phi ) & =\sum_{i\in I_{1}}w_{i}\cos^{q_{1}}\theta \;\nu_{i}\left(\theta ,\phi \right)\;+\;\sum_{i\in I_{2}}w_{i}\cos^{q_{2}}\theta \;\nu_{i}\left(\theta ,\phi \right)\\ \; & =\cos^{q_{1}}\theta \;w_{1}^{H}\nu_{1}\left(\theta ,\phi \right)+\cos^{q_{2}}\theta \;w_{2}^{H}\nu_{2}\left(\theta ,\phi \right) \end{array}$$

where $w_{1},w_{2}$ and $\nu_{1},\nu_{2}$ collect the weights and manifold values of elements of type 1 and 2, respectively.

### 2.2. Directivity for Two Radiating Element Classes

Two radiating element classes can be estimated with two sizes of radiating elements. Deriving the formulation from [7], it is possible to link the size of one circular radiating element aperture with *q*. The values of *q* can therefore be estimated thanks to Equation (3).

(3) $$q=2.07{\left(\frac{d}{\lambda }\right)}^{2}-0.5$$

where *d* denotes the inter-element spacing. It is noted that *q* increases proportionally with larger dimensions.

The directivity at look direction $\left(\theta_{0},\phi_{0}\right)$ is defined in Equation (4).

(4) $$D\left(\theta_{0},\phi_{0}\right)=\frac{|f\left(\theta_{0},\phi_{0}\right){|}^{2}}{\frac{1}{4\pi }\int_{0}^{2\pi }\;\int_{0}^{\pi }{\left|f\left(\theta ,\phi \right)\right|}^{2}\sin\theta \;d\theta \;d\phi }.$$

From (2),

(5) $$\begin{array}{cc} |f\left(\theta_{0},\phi_{0}\right){|}^{2}= & \cos^{2q_{1}}\theta_{0}\;|w_{1}^{H}\nu_{1}\left(\theta_{0},\phi_{0}\right){|}^{2}+\cos^{2q_{2}}\theta_{0}\;{\left|w_{2}^{H}\nu_{2}\left(\theta_{0},\phi_{0}\right)\right|}^{2}\\ \; & \;+2ℜ\;\left\{\cos^{q_{1}+q_{2}}\theta_{0}\;\left(w_{1}^{H}\nu_{1}\right){\left(w_{2}^{H}\nu_{2}\right)}^{*}\right\}. \end{array}$$

The three terms correspond to radiation from class-1 elements, radiation from class-2 elements, and the mixed interference term weighted by $\cos^{q_{1}+q_{2}}\theta_{0}$. This mixed term is the key feature of multi-*q* arrays and will drive the structure of the directivity denominator.

The radiated power is defined in Equation (6).

(6) $$P_{\mathrm{rad}}=\frac{1}{4\pi }\int {\left|f\left(\theta ,\phi \right)\right|}^{2}d\Omega ,$$

which expands as Equation (7).

(7) $$\begin{array}{cc} P_{\mathrm{rad}} & =w_{1}^{H}B_{11}w_{1}+w_{2}^{H}B_{22}w_{2}+2ℜ\left\{w_{1}^{H}B_{12}w_{2}\right\}, \end{array}$$

where block matrices $B_{11},B_{22},B_{12}$ are defined elementwise as Equation (8).

(8) $$\begin{array}{cc} {\left[B_{nm}\right]}_{i,k} & =\frac{1}{4\pi }\int_{0}^{2\pi }\;\int_{0}^{\pi }\cos^{q_{n}+q_{m}}\theta \;e^{jk\Delta r_{ik}\sin\theta \cos\left(\phi -\phi_{ik}\right)}\\ \; & \;\;\;\times \sin\theta \;d\theta \;d\phi , \end{array}$$

with inter-element projected spacing $\Delta r_{ik}=\sqrt{{\left(x_{i}-x_{k}\right)}^{2}+{\left(y_{i}-y_{k}\right)}^{2}}.$

Evaluating the azimuth integral produces a Bessel function, leading to the closed form in Equation (9).

(9) $$\begin{array}{cc} {\left[B_{nm}\right]}_{i,k} & =2^{\frac{q_{n}+q_{m}}{2}-\frac{3}{2}}\Gamma \;\left(\frac{q_{n}+q_{m}+1}{2}\right)\frac{J_{\frac{q_{n}+q_{m}+1}{2}}\left(k\Delta r_{ik}\right)}{{\left(k\Delta r_{ik}\right)}^{\frac{q_{n}+q_{m}+1}{2}}}. \end{array}$$

The dependence on $q_{n}+q_{m}$ reflects the coupling between element types. This leads to a quadratic form in the weights for the radiated power.

Collect the weight vectors into $w=\left[\begin{array}{c} w_{1}\\ w_{2} \end{array}\right],B=\left[\begin{array}{cc} B_{11} & B_{12}\\ B_{12}^{H} & B_{22} \end{array}\right].$

Similarly define the numerator steering vector $a\left(\theta_{0},\phi_{0}\right)=\left[\begin{array}{c} \cos^{q_{1}}\theta_{0}\;\nu_{1}\left(\theta_{0},\phi_{0}\right)\\ \cos^{q_{2}}\theta_{0}\;\nu_{2}\left(\theta_{0},\phi_{0}\right) \end{array}\right].$

Then the directivity is compactly given by Equation (10).

(10) $$D\left(\theta_{0},\phi_{0}\right)=\frac{{\left|w^{H}a\left(\theta_{0},\phi_{0}\right)\right|}^{2}}{w^{H}Bw}.$$

### 2.3. Generalization to Q Classes of $\cos^{q}$ Radiating Elements

Now assume each element belongs to one of *Q* types with patterns $E_{n}\left(\theta \right)=\cos^{q_{n}}\theta ,\;n=1,\ldots ,Q.$

Let $I_{n}$ denote the set of elements of type *n*, with weight vector $w_{n}$ and manifold vector $\nu_{n}\left(\theta ,\phi \right)$.

The array factor generalizes to Equation (11).

(11) $$f\left(\theta ,\phi \right)=\sum_{n=1}^{Q}\cos^{q_{n}}\theta \;w_{n}^{H}\nu_{n}\left(\theta ,\phi \right).$$

The numerator becomes Equation (12).

(12) $$f\left(\theta_{0},\phi_{0}\right){|}^{2}=\sum_{n=1}^{Q}\sum_{m=1}^{Q}\cos^{q_{n}+q_{m}}\theta_{0}\;w_{n}^{H}\nu_{n}\nu_{m}^{H}w_{m}.$$

The radiated power is Equation (13).

(13) $$P_{\mathrm{rad}}=\sum_{n=1}^{Q}\sum_{m=1}^{Q}w_{n}^{H}B_{nm}w_{m},$$

where each $B_{nm}$ is given by the same integral structure as (8) with exponent $q_{n}+q_{m}$, admitting the same closed form (9).

Collecting all weights, $w=\left[\begin{array}{c} w_{1}\\ \vdots \\ w_{Q} \end{array}\right],\;B=\left[\begin{array}{ccc} B_{11} & \cdots & B_{1Q}\\ \vdots & \ddots & \vdots \\ B_{Q1} & \cdots & B_{QQ} \end{array}\right],$ anddefining the numerator steering vector $a\left(\theta_{0},\phi_{0}\right)=\left[\begin{array}{c} \cos^{q_{1}}\theta_{0}\;\nu_{1}\\ \vdots \\ \cos^{q_{Q}}\theta_{0}\;\nu_{Q} \end{array}\right],$ the directivity is Equation (14).

(14) $$D\left(\theta_{0},\phi_{0}\right)=\frac{|w^{H}a\left(\theta_{0},\phi_{0}\right){|}^{2}}{w^{H}Bw}.$$

Assume that for Q=n radiating element classes we have the valid directivity expression of Equation (15).

(15) $$D_{n}=\frac{|w^{(n)H}a^{\left(n\right)}{|}^{2}}{w^{(n)H}B^{\left(n\right)}w^{\left(n\right)}}$$

with the n×n block matrix $B^{\left(n\right)}$ defined in the same manner as above.

We introduce radiating element type n+1 with pattern $\cos^{q_{n+1}}\theta$. The numerator obtains the additional term $\cos^{q_{n+1}}\theta_{0}\;w_{n+1}^{H}\nu_{n+1}.$

The denominator gains:

- A new self-block $B_{n+1,n+1}$;
- Cross-blocks $B_{n+1,r}$ and $B_{r,n+1}$ for each existing class *r*.

Each is constructed by replacing $q_{n}+q_{m}$ with $q_{n+1}+q_{r}$ in Equations (8) and (9).

Therefore the augmented matrix becomes $B^{\left(n+1\right)}=\left[\begin{array}{cc} B^{\left(n\right)} & C\\ C^{H} & B_{n+1,n+1} \end{array}\right],$ where *C* collects all $B_{r,n+1}$ cross-blocks.

Because the integral structure depends only on the exponent $q_{r}+q_{n+1}$ and the projected spacing $\Delta r_{ik}$, the mathematical form of the *B*-blocks is preserved. Therefore, the same quadratic-form expression for directivity remains valid.

Since the structure holds for n=1 when calculating by recursion, and remains invariant when adding a new element type, the directivity formula (14) is valid for all Q≥1.

#### Normalization of the Directivity

As shown in [21], the radiated power associated with a $\cos^{q}\theta$ embedded element pattern must be properly normalized to ensure that the total radiated power remains constant across different element classes. For a single class of radiating elements with exponent *q*, the corresponding normalization factor is Equation (16).

(16) $$D\left(\cos^{q}\theta \right)=2\left(2q+1\right),$$

which guarantees that the integrated power pattern over the sphere matches the reference level.

This expression can be extended by recursion to arrays composed of *Q* distinct classes of radiating elements. Let $n_{i}$ denote the number of elements of class *i*, with total array size *N*, and let each class be characterized by an exponent $q_{i}$. The generalized normalization factor becomes Equation (17).

(17) $$D\left(\cos\theta \right)=2\left(2\sum_{i=1}^{Q}\frac{n_{i}}{N}q_{i}+1\right).$$

This normalization prevents an artificial increase in total radiated power as the exponents $q_{i}$ grow, ensuring a physically consistent directivity evaluation and enabling fair comparison of array architectures employing different element types.

## 3. Results

### 3.1. Validation of the Technique on Linear Arrays

**Figure 1:**
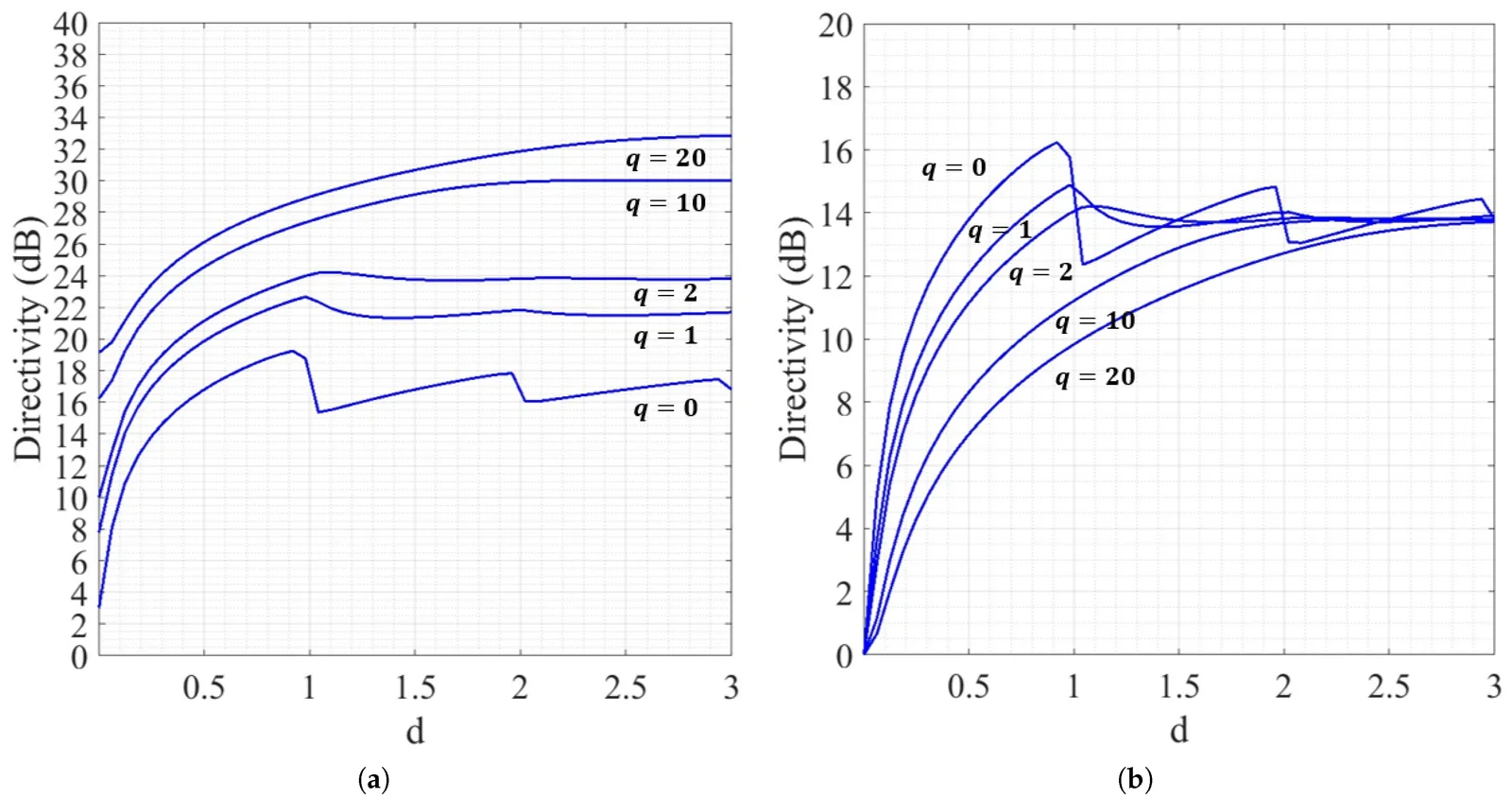
Directivity for a linear array of 24 elements with different *q*, fixed at first across the array: (**a**) Not normalized directivity, using no D(cosθ) boundary; (**b**) normalized patterns.

The validity of the proposed formulation and its associated normalization procedure has been verified by comparing the obtained results with those reported in [21]. This allows benchmarking the model for both the case of identical radiating elements (same *q*) and heterogeneous configurations involving different *q* values. The first validation case in Figure 1a considers a linear array in which all radiating elements share the same physical size. In this comparison, the element size is kept constant and does not depend on *q*, ensuring that variations in the radiation characteristics arise solely from the $\cos^{q}\theta$ model. Starting with isotropic radiating elements (q=0), directivity values were evaluated for different numbers of elements and varying inter-element spacings *d*. The results accurately reproduce those from Figures 3 and 4 in [21]. confirming the correctness of the formulation with the baseline isotropic case.

A second validation concerns arrays composed of two distinct element classes with different exponents *q*. After applying the generalized normalization derived previously, the resulting directivity values are again in agreement with those in [21]. For instance, in a linear array of 24 radiating elements, the directivity is observed to converge toward approximately 13.8 for large inter-element spacings (here, d=3), consistent with the asymptotic behavior reported in the reference. It is noted that instead the non-normalized pattern converges to a maximum directivity of 16 dB approximately. Moreover, for isotropic radiating elements, periodic drops in directivity occur as the spacing increases, reflecting the expected effects of grating-lobe formation [26]. In contrast, cases with q≠0 exhibit smoother directivity curves, since the broader or tapered element patterns reduce the oscillatory behavior induced by the array factor. These results confirm that the proposed formulation and normalization correctly reproduce established behaviors for both uniform and heterogeneous element classes.

### 3.2. Validation Against Full-Wave Analysis Tool

Populating an array aperture with different-size radiating elements is of particular interest in case (a) the array aperture is electrically quite large or (b) the field of view is limited so even medium/large radiating elements (with associated constraints in terms of grating lobes) can be considered. As is well known, the coupling coefficients between neighbours elements are particularly limited for medium/large radiating elements. So especially in these conditions, the analytical procedure to estimate the directivity could be useful in the preliminarily phase of the design. In order to test the validity of our solution, we consider an example from the literature [16]. A discrete lens antenna populated by 218 radiating elements is proposed, with different sizes of radiating elements. The results reported in [16] are based on full-wave analysis and therefore provide a good reference baseline. In addition, the computation time is specified, together with the methodology used to derive the radiation pattern and directivity. Starting from this reference, the $\cos^{q}$ directivity formulation has been benchmarked in terms of radiation pattern shape and directivity level.

**Figure 2:**
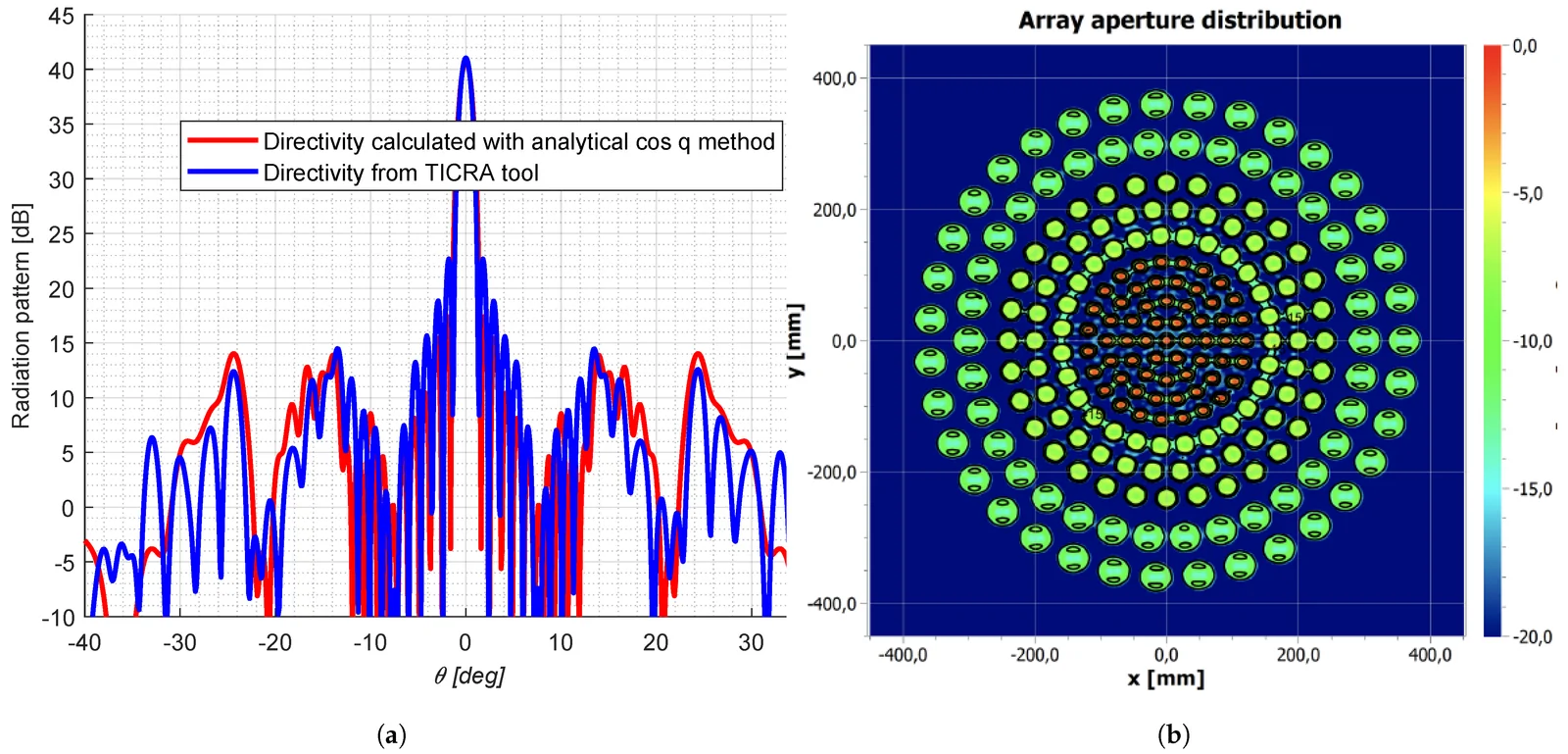
(**a**) Radiation patterns obtained from the configuration in [16] with 218 radiating elements and with the $\cos^{q}$ directivity calculation. (**b**) Reference architecture taken from Figure 4 in [16].

In Figure 2 the full-wave analysis results derived with the Ticra tool [27] are compared with the patterns evaluated with the analytical procedure presented here for the first time. As is evident, the agreement between the two solutions is very good and this demonstrates the validity of our solution. Both the pattern shape and the directivity level are extremely well aligned.

In terms of running time, it is specified in [16] that the full-wave analysis can take about 10 min on an Intel Core i7-13850HX 2.1 GHz with a 20-core laptop. In comparison, the formulation based on the analytical $\cos^{q}$ calculation runs in less than a second (0.35 s), demonstrating a significant improvement.

### 3.3. Application to Ring Arrays

The architectures presented in [24] rely on a deterministic synthesis approach in which both the positions and the dimensions of the radiating elements are jointly optimized to overcome the aperture-efficiency limitations inherent to sparse arrays with identical elements. The method begins by approximating a continuous circular aperture distribution through a set of maximum-efficiency annular rings exhibiting stepwise constant radial field profiles. These continuous annuli are subsequently discretized into maximum-efficiency radiating elements—typically circular apertures with diameters proportional to the width of their corresponding annular ring—thereby minimizing unused aperture area and maximizing the filling factor. Several array configurations are considered, including amplitude-tapered annular-ring arrays, equi-amplitude arrays with identical radiating elements, and architectures in which both element density and element size are tapered to better approximate the desired aperture while preserving high RF power efficiency. The synthesis framework also accommodates constraints on maximum element dimensions to satisfy beam-scanning requirements, making the resulting arrays suitable for wide-coverage phased-array applications. Because the achievable directivity differs from that of the ideal continuous aperture only by the intrinsic filling-factor loss of circular annular arrangements (approximately 1.05 dB), these architectures constitute a robust reference baseline against which cosq element types can be introduced and evaluated. Moreover, the procedure is general and supports alternative maximum-efficiency element geometries—such as circular, square, or annular-sector apertures—allowing new $\cos^{q}$ element implementations to be integrated seamlessly into the directivity optimization process.

#### 3.3.1. Comparison Between the Two Ring Architectures

In the following, we aim at comparing the two unconstrained architectures from [24], defined as follows. In the unconstrained size-tapered configuration employing 128 radiating elements, the reference continuous aperture is partitioned into a set of elementary annuli through inversion of the grading function, leading to approximately ten annular regions of increasing radial width. Each annulus is subsequently replaced by a ring of maximum-efficiency circular radiating elements whose diameter equals the width of the corresponding annulus, resulting in element sizes that grow from about 2λ at the center to nearly 3.4λ at the periphery. The 128-element solution arises when the mid-radius of each ring is chosen as the geometric center of the elementary annulus, which produces slightly wider annuli in the outer portion of the aperture and therefore reduces the number of elements computed on those rings according to $N_{m}=\left\lfloor 2\pi \rho_{m}/d_{m}\right\rfloor$. This yields a characteristic density taper, with element populations increasing from a single central element to more than twenty elements on the largest rings, while maintaining equi-power excitation across all elements. The resulting architecture achieves an aperture-efficiency loss of approximately 1.55 dB, which is very close to the theoretical 1.05 dB filling-factor limit for circular sparse arrays, confirming that jointly optimizing element density and diameter provides excellent control of the radiation pattern with a substantially reduced number of radiating elements.

A modified variant of the unconstrained sparse architecture is obtained by enforcing a reduction of the element diameter on the outermost ring so that it matches the diameter of the second outer ring. In this configuration, all annuli up to the second outer ring follow the unconstrained size-tapering law, generating increasing element diameters proportional to annulus width. However, instead of allowing the outermost annulus to grow to its natural width (and thus produce the largest elements of roughly 3.4λ), its element diameter is constrained to the smaller value associated with the second outer ring. This adjustment reduces the radial width of the final annulus and increases the number of elements on the outer ring via $N_{m}=\left\lfloor 2\pi \rho_{m}/d_{m}\right\rfloor$, effectively shifting part of the aperture control from size tapering to density tapering. The resulting array maintains the desirable property of progressively increasing density toward the periphery while ensuring smoother element-size transitions between rings, which can be advantageous for manufacturing or for limiting element-factor variation. Although this constraint slightly reduces the maximum achievable aperture filling factor, the architecture preserves the low sidelobe performance characteristic of the size- and density-tapered approach while offering a more uniform element-size distribution across the outer portion of the array.

#### 3.3.2. Radiation Patterns

**Figure 3:**
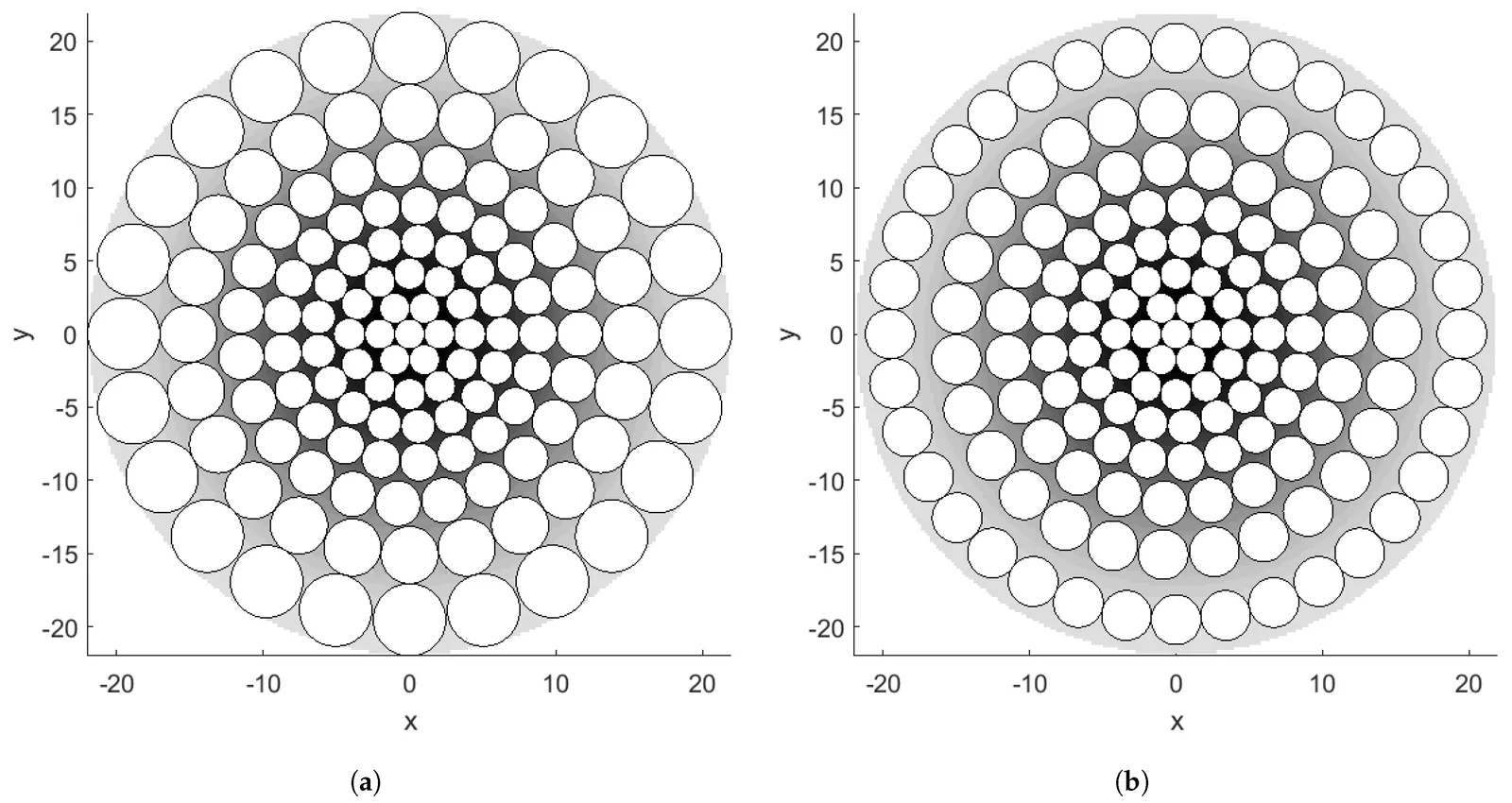
Architecture definition with $\cos^{q}$ elements. (**a**) Case 1: Continuously tapered array with associated increase of the element size; (**b**) case 2: taper identical to case 1 but with outer ring populated with smaller elements.

The architectures are summed up in Table 1. The normalized radiation patterns are presented in Figure 3. As shown, first, the sidelobes are positioned the same as per the maximum-efficiency (ME) elements. Secondly, for case 1, it is noticed that the first-order sidelobe is slightly lower for the $\cos^{q}$ case, while sidelobes further to the main lobe are increased. Overall, the sidelobe floor average is equivalent between the two cases. Noticeably, the beamwidth is slightly increased by 0.012. For case 2, the sidelobes at the edges are reduced while the other sidelobes are increased. Overall, the level of sidelobes differs from ME to $\cos^{q}$ element patterns, but the same characteristic still occurs, which validated the normalized pattern calculation.

#### 3.3.3. Directivity

We now evaluate the directivity obtained using the defined $\cos^{q}$ element formulation and compare it with the theoretical upper bound. The maximum achievable directivity for the considered apertures can be inferred from the continuous circular area they occupy. With the outer ring radius set to 18.5 m, the corresponding geometric limit is approximately 37.53 dB.

Because the radiating elements are circular, part of the aperture area remains unused. In case 1, the effective radiating area corresponds to 58.9% of the total continuous aperture, leading to an expected directivity reduction of about 2.3 dB due to area loss. In case 2, the coverage is further reduced to 52.0%, primarily because of the smaller elements located near the periphery that introduce additional spacing between the two outer rings. This results in an anticipated loss of approximately 2.79 dB.

The resulting directivity values are summarized in Table 1. As shown, the degradation associated with the $\cos^{q}$ elements closely matches the theoretical loss predicted from the covered-area ratio. Consequently, the directivity reduction is more pronounced in case 2 than in case 1.

**Figure 4:**
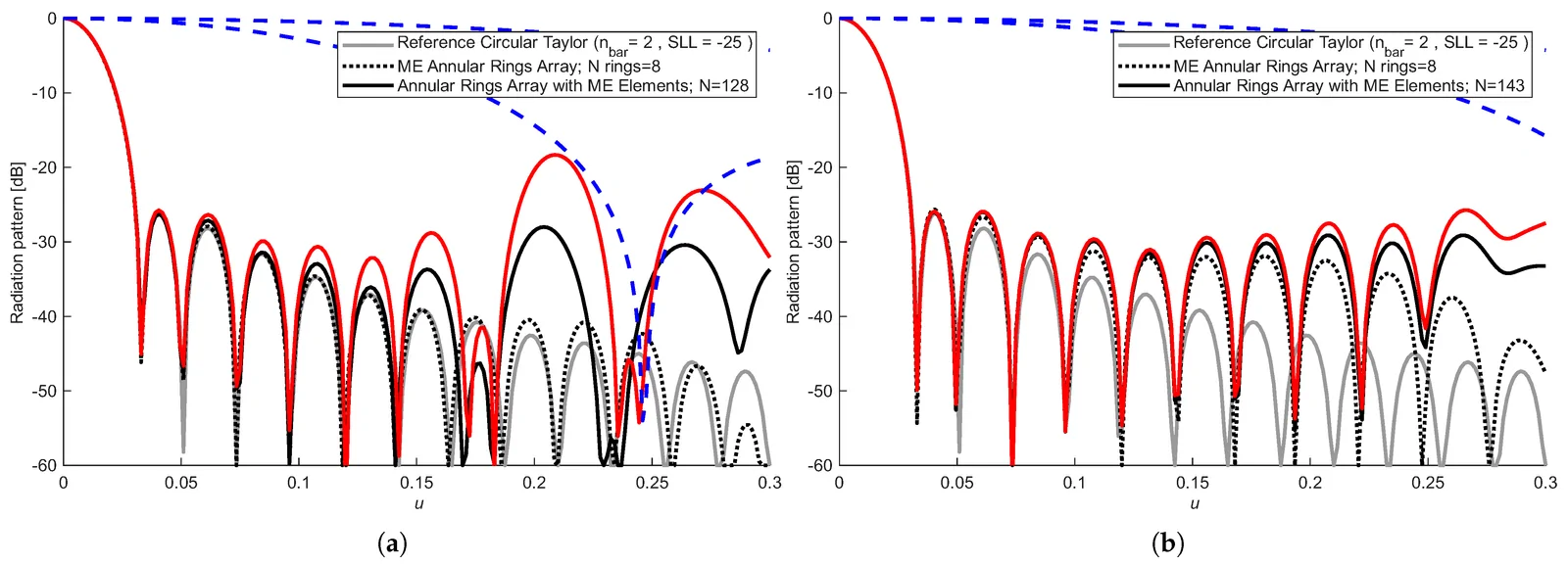
Radiation patterns normalized to 0 dB for the Reference Circular Taylor with −25 dB target (grey), ME Annular Ring Array (dot lines), Annular Ring Array with ME Elements (black) and Annular Ring Array with $\cos^{q}$ elements (red). (**a**) Case 1: Continuously tapered array with associated increase of the element size; (**b**) Case 2: taper identical to Case 1 but with outer ring populated with smaller elements.

Two main conclusions can be drawn:

- Higher directivity in Case 1: The radiating elements provide better aperture coverage and therefore achieve a larger effective area. In contrast, case 2 exhibits a coverage gap between the outer rings, which slightly decreases the directivity.
- Sidelobe behavior: Case 1 exhibits overall larger and higher sidelobes, whereas case 2 offers improved sidelobe control, particularly near the edges of the coverage region. This trend is consistent with the observations reported in [24]. This can be observed in Figure 4.

## 4. Discussion

In this paper, the directivity of rings arrays with tapered sizes with $\cos^{q}$ elements has been investigated. The directivity evaluation enables a fast modeling for LEO or MEO arrays, which is important for fast communications, navigation. The solution voids the consideration of the integral. The mathematics have been verified by using the linear array case introduced in the literature. The results were in line with what has been previously demonstrated. As compared with results obtained using full-wave analysis, the radiation pattern derived from the $\cos^{q}$ method is significantly faster to compute while maintaining very good agreement. The residual discrepancy with respect to the full-wave reference originates from the field approximation used in the $\cos^{q}$ formulation. Extending the case to planar arrays, the paper also demonstrates that the formula is applicable to an arbitrary number of $\cos^{q}$ elements. Two practical cases have been investigated. Both of them are based on ring arrays with tapered size radiating elements. The results have been checked as compared to theoretical geometrical results in terms of directivity, and sidelobe level distribution. The analysis done also represents a validation of the heuristic procedure to associate a q factor with a radiating element with an assigned aperture.

## Figures and Tables

**Table 1:** Directivity calculation summary table.

Architecture	Directivity	Difference with the Maximum Area	Difference with Theoretical Maximum Directivity	Sidelobe Level
Case 1	35.23 dB	58.9%	−2.30 dB	−19.72 dB
Case 2	34.72 dB	52.0%	−2.79 dB	−27.67 dB

## Data Availability

Data available on request due to restrictions.

## Abbreviations

The following abbreviations are used in this manuscript:

EEP	Embedded Element Pattern
FFT	Fast Fourier Transform
LEO	Low Earth Orbit
ME	Maximum Efficiency
MEO	Medium Earth Orbit
